# Safety and efficacy of canine recombinant IL-15 in mammary gland tumors

**DOI:** 10.3389/fvets.2025.1603421

**Published:** 2025-06-09

**Authors:** Min-Hee Kang, Jaeil Lee, Sang-Ki Kim, Kyeyoung Koh, Mi-Ae Kang, Hee-Myung Park

**Affiliations:** ^1^Department of Bio-animal Health, Jangan University, Gyeonggi-do, Republic of Korea; ^2^Biomaterial R&BD Center, VaxCell-Bio, Gwangju, Republic of Korea; ^3^Vet&Gene, Seongnam, Republic of Korea; ^4^Department of Veterinary Internal Medicine, College of Veterinary Medicine, Konkuk University, Seoul, Republic of Korea

**Keywords:** canine mammary tumors, recombinant IL-15, immunomodulation, antitumor efficacy, safety assessment

## Abstract

This study investigated the safety and therapeutic potential of recombinant canine interleukin-15 (rcIL-15) as an adjunct to surgical excision in dogs with mammary gland tumors (MGTs). Sixty-one client-owned dogs were initially enrolled, and 55 completed the 12-week study. Dogs were assigned to a test group receiving rcIL-15 with surgery or a control group undergoing surgery alone. RcIL-15 was administered intravenously at 20 μg/kg/day for two 4-day cycles, separated by a 10-day rest period. Clinical monitoring included physiological assessments, blood tests, and owner-reported quality of life (QoL) questionnaires. No significant adverse changes were observed in hematological, biochemical, or physiological parameters. RcIL-15 was well tolerated, with only mild and self-limiting gastrointestinal signs and injection site reactions. Dogs in the rcIL-15 group showed significantly improved QoL, particularly in pain, appetite, and activity. Notably, serum C-reactive protein levels decreased over time in the rcIL-15 group, suggesting reduced systemic inflammation. VEGF levels remained stable in the test group but increased in controls, while IFN-*γ* concentrations rose significantly following rcIL-15 treatment, indicating immune activation. These findings suggest that rcIL-15 may enhance antitumor immunity and alleviate tumor-related inflammation without compromising safety. The combination of surgery and rcIL-15 improved clinical outcomes compared to surgery alone, supporting its use as a novel immunotherapeutic adjunct for canine MGTs. However, limitations include the short follow-up period and lack of cellular immune profiling. Further studies with longer follow-up and immunological assessment are warranted to confirm these results and explore the broader application of rcIL-15 in veterinary oncology.

## 1 Introduction

Cancer is a leading cause of mortality in dogs, with mammary gland tumors (MGTs) being the most frequently diagnosed neoplasm in intact female dogs worldwide (1, 2). MGTs account for approximately 14.5% of all canine tumors, with a significant proportion being malignant (3). The incidence of malignant MGTs varies by region, reflecting differences in reproductive management practices. In countries where ovariohysterectomy is not routinely performed, such as many European nations, the incidence of MGTs is notably higher compared to regions where early spaying is common (2, 4). Intact female dogs are at a significantly higher risk of developing MGTs, with the risk increasing with each estrous cycle before spaying (1). Beyond hormonal factors, breed predisposition and genetic susceptibility are important risk determinants, with certain breeds such as Toy Poodles, English Springer Spaniels, and Cocker Spaniels being particularly prone to MGTs (3). Human breast cancer shares similar features, including hormonal dependence, molecular alterations such as p53 and HER2/neu expression, and a wide spectrum of histopathological subtypes (2). These findings suggest that MGTs in dogs share several biological and clinical characteristics with human breast cancer, making them valuable models for comparative oncology research. In addition, rodent mammary tumor models have shown histopathological and receptor-expression similarities to human breast cancer, further supporting cross-species comparative approaches (5).

Despite the high incidence, treatment options for canine mammary gland tumors are primarily limited to surgical excision, with few veterinary-specific anticancer drugs available. Current therapeutic strategies rely mainly on surgical intervention, ranging from lumpectomy to radical mastectomy, often guided by lymphatic drainage patterns (4). However, the optimal surgical approach remains controversial due to inconsistent evidence on the impact of surgical margins and extent on long-term outcomes. While radical mastectomy is traditionally preferred for malignant tumors to minimize recurrence risk, it is associated with increased postoperative complications (6). In contrast, less invasive approaches have shown comparable survival outcomes in some studies but carry a higher risk of local recurrence (7, 8). Moreover, the role of adjuvant therapies, including chemotherapy and hormone therapy, remains poorly defined in veterinary oncology, highlighting the need for innovative therapeutic strategies.

Immunotherapy has emerged as a promising modality in cancer treatment due to its potential to enhance anti-tumor immunity while minimizing systemic toxicity (9). Among immunotherapeutic agents, recombinant interleukin-15 (IL-15) is particularly promising due to its potent stimulation of natural killer (NK) cells and CD8 + T cells, which are crucial for anti-tumor immunity (10). IL-15 has been shown to promote the survival and proliferation of these effector cells, enhancing their cytotoxic activity against tumor cells (11). These unique immunostimulatory properties position IL-15 as a potential therapeutic candidate for enhancing anti-tumor immunity in canine MGTs.

Based on these mechanisms, we hypothesize that recombinant canine IL-15 (rcIL-15), when administered as an adjunct to surgical excision, can improve clinical outcomes, enhance immune activation, and improve quality of life in dogs with mammary gland tumors. This study was therefore designed to evaluate the safety and efficacy of rcIL-15 in this context.

## 2 Materials and methods

### 2.1 Study design and ethical approval

This clinical trial was conducted to evaluate the safety and efficacy of rcIL-15 as an adjunct therapy for dogs diagnosed with mammary gland tumors. The study was approved by the Institutional Animal Care and Use Committee (Approval Number: CPT-23-015-M) and conducted according to ethical guidelines for animal research. All procedures adhered to the ethical standards for veterinary clinical trials, and informed consent was obtained from all dog owners before enrollment. All procedures were designed to minimize discomfort and stress for the dogs, and humane endpoints were established to safeguard animal welfare. Dogs were monitored closely for adverse events, and veterinary care was provided as needed. The study was conducted from January 6, 2023, to September 30, 2023.

### 2.2 Study population and eligibility criteria

Dogs diagnosed with mammary gland tumors were recruited from 18 veterinary hospitals in South Korea between January 2023 and September 2023. A total of 61 client-owned dogs were enrolled, and 55 dogs completed the study. Eligible dogs were required to have a diagnosis of mammary gland tumors confirmed by either cytology or histopathology, and to be in sufficient health to undergo surgical excision and participate in the study. Dogs were excluded if they had concurrent malignant tumors unrelated to mammary gland tumors, severe systemic illnesses, or comorbidities that could interfere with the evaluation of study outcomes. Additional exclusion criteria included pregnancy, lactation, enrollment in other clinical trials, or the presence of conditions known to impair immunological responses. Dogs with no prior chemotherapy or immunotherapy within the last 3 months were included to minimize confounding factors. Dogs were randomly assigned to either the test group receiving rcIL-15 with surgical excision or the control group undergoing surgical excision alone using a simple randomization method. While group allocation was not stratified by breed, body weight, or age, baseline characteristics were reviewed to ensure general balance between groups. This study was conducted as an open-label trial; both owners and attending veterinarians were aware of group assignments. However, all laboratory-based assessments, including hematology, serum chemistry, and biomarker analyses, were performed in a blinded fashion to minimize measurement bias.

### 2.3 Treatment protocol

The test group received rcIL-15 in combination with surgical excision, while the control group underwent surgical excision alone. The rcIL-15 was manufactured using *E. coli* BL21(DE3) and pET30a(+) vector, yielding a clear injectable solution stored in 1 mL transparent glass vials at 2–8°C. The administration protocol involved intravenous infusion at a dose of 20 μg/kg/day for four consecutive days, followed by a 10-day rest interval. This cycle was repeated once more. This fixed dosing strategy was uniformly applied based on body weight, without further individual dose adjustments. The selected dosage and schedule were based on previous optimization studies in healthy dogs, which demonstrated that repeated administration of rcIL-15 at this level effectively activated cytotoxic immune cells, such as NK and CD8 + T cells, without inducing serious adverse effects (12, 13). Each dose was diluted in a small volume of normal saline and delivered slowly over 10 min through an indwelling intravenous catheter. Surgical removal of mammary gland tumors was conducted using standardized procedures, and postoperative care, including pain management and wound care, was administered according to clinical guidelines. No chemotherapeutic agents were used during the study to isolate the effects of rcIL-15.

### 2.4 Clinical assessments

Clinical assessments were conducted at baseline (pre-treatment) and at 2-, 4-, 8-, and 12-week post-treatment to monitor general health status and detect any adverse events. General observations included medication history, patient history, and clinical interviews to assess recent illnesses, surgeries, and clinical signs. Physical examinations were conducted during each visit to measure body weight, body condition score (BCS, 1–9 scale), vital signs (body temperature, heart rate, respiratory rate), and systolic blood pressure. Owner-reported quality of life was assessed using validated questionnaires (14), evaluating appetite, activity levels, hydration, and overall well-being.

Imaging studies, including radiography, ultrasound, and CT scans, were performed when further evaluation was needed. Hematological assessments comprised complete blood count (CBC), blood chemistry, and electrolyte tests. Blood samples were stored at −20°C until analysis and processed in duplicate to ensure reliability. Clinical signs and laboratory results were systematically recorded, and potential associations with rcIL-15 administration were evaluated by attending veterinarians.

### 2.5 Efficacy and safety evaluation

Efficacy was evaluated by comparing inflammation and thrombosis markers (CRP and D-dimer) and tumor-related biomarkers (VEGF and IFN-*γ*) between the test and control groups. Quality of life (QoL) assessments were conducted using owner-reported questionnaires (14), and clinical symptom improvement was evaluated by veterinarians.

Safety was assessed by monitoring hematological and clinical chemistry parameters for abnormalities that could indicate adverse effects. Vital signs, including body weight, body temperature, heart rate, and respiratory rate, were regularly monitored to detect any side effects. Side effects were categorized according to the Veterinary Cooperative Oncology Group-Common Terminology Criteria for Adverse Events (VCOG-CTCAE v2) to ensure consistent reporting and severity grading (15). All side effects were managed following standard veterinary care guidelines, and no cases required discontinuation of rcIL-15 therapy.

### 2.6 Statistical analysis

Data were expressed as mean ± standard deviation (SD) or mean ± standard error (SE). The Kolmogorov–Smirnov test was used to assess the normality of data distribution. For normally distributed data, Student’s t-test was used to compare continuous variables between the test and control groups, while the Mann–Whitney U test was applied for non-normally distributed data. Within-group comparisons before and after rcIL-15 administration were evaluated using paired t-tests or Wilcoxon signed-rank tests, depending on data distribution. Repeated measures ANOVA was used to analyze the effects of time, group, and time-by-group interactions. For categorical variables, Pearson’s chi-square test was used to determine statistical significance. Statistical significance was set at *p* < 0.05. Data analysis was performed using SPSS version 20 (SPSS Inc., Chicago, IL, United States) and GraphPad Prism version 9.5.1 (GraphPad Software Inc., Boston, MA, United States).

## 3 Results

### 3.1 Baseline characteristics of dogs

A total of 61 client-owned dogs diagnosed with mammary gland tumors were enrolled in this clinical trial. Among them, 55 dogs completed the study, while six were excluded due to either withdrawal (*n* = 4; one in the control group and three in the test group) or death during the trial period (*n* = 2; one in each group). Table 1 presents the baseline characteristics of the dogs that completed the study.

**Table 1:** Baseline characteristics of enrolled dogs in the MGT clinical trial.

Characteristic	Test group (*n* = 27)	Control group (*n* = 28)
Age (years)	11.5 ± 2.9	8.9 ± 2.8
Weight (kg)	4.2 ± 1.9	9.8 ± 8.4
Sex (n)		
Female	2	11
Spayed Female	25	17
Breed (n)	Maltese (9), Yorkshire Terrier (4), Poodle (3), Dachshund (3), Shih Tzu (3), Mixed Breed (2), Pomeranian (2), Spitz (1)	Mixed Breed (11), Maltese (3), Poodle (2), Pomeranian (2), Shih Tzu (2), Jindo Dog (2), Yorkshire Terrier (1), Miniature Pinscher (1), Cocker Spaniel (1), Spitz (1), Dachshund (1), Bullmastiff (1)

Age and weight values are presented as mean ± standard deviation. MGT, mammary gland tumor.

The mean age of the dogs was 11.5 ± 2.9 years in the test group and 8.9 ± 2.8 years in the control group, with most dogs being over 7 years old, reflecting the prevalence of mammary gland tumors in aging pets. The majority of the dogs were small breeds, including Maltese, Poodle, Yorkshire Terrier, and Shih Tzu, which aligns with common pet ownership trends in South Korea. In the control group, a few larger breeds such as Jindo and Bullmastiff were included, resulting in a broader weight range (9.8 ± 8.4 kg) compared to the test group (4.2 ± 1.9 kg).

Tumor staging was determined using the TNM classification system (16) and grouped into five stages based on tumor size, lymph node involvement, and distant metastasis (Table 2). In the test group (*n* = 27), the distribution was: Stage 1 (*n* = 14), Stage 2 (*n* = 4), Stage 3 (*n* = 4), Stage 4 (*n* = 2), and Stage 5 (*n* = 3). In the control group (*n* = 28), the staging distribution was: Stage 1 (*n* = 18), Stage 2 (*n* = 1), Stage 3 (*n* = 2), Stage 4 (*n* = 4), and Stage 5 (*n* = 3). There was no significant difference in TNM stage distribution between the two groups (*p* = 0.520).

**Table 2:** Tumor stage distribution based on TNM classification.

Tumor stage^a^	Test group (*n* = 27)	Control group (*n* = 28)	*p*-value
Stage 1	14	18	0.520
Stage 2	4	1	
Stage 3	4	2	
Stage 4	2	4	
Stage 5	3	3	

a Tumor staging was based on the TNM (Tumor, Node, Metastasis) classification system, where Stage 1 corresponds to T1N0M0 (tumor ≤3 cm, no regional lymph node involvement, no distant metastasis); Stage 2: T2N0M0 (tumor >3 cm and ≤5 cm); Stage 3: T3N0M0 (tumor >5 cm); Stage 4: any T, N1M0 (regional lymph node involvement present); and Stage 5: any T, any N, M1 (distant metastasis present).

### 3.2 Physiological parameters

To evaluate the physiological effects of rcIL-15, six vital parameters were monitored, including body weight, body condition score (BCS), body temperature, heart rate, respiratory rate, and systolic blood pressure. Measurements were taken at five time points: baseline (pre-treatment), and at 2, 4, 8, and 12 weeks post-treatment. Statistical analyses were performed to assess within-group changes over time and between-group differences at each time point (Table 3).

**Table 3:** Changes in vital parameters in test and control groups with mammary gland tumors.

Vital parameters	Groups	0 W	2 W	4 W	8 W	12 W	*p* (time)	*p* (group)
Body weight (Kg)	Test	4.3 ± 1.9	4.3 ± 1.9	4.3 ± 1.8	4.1 ± 1.7	4.1 ± 1.6	0.355	0.002*
	Control	9.8 ± 8.4	9.8 ± 8.4	9.7 ± 8.3	9.7 ± 8.3	9.7 ± 8.3		
BCS (5 scale)	Test	3.1 ± 0.7	3.1 ± 0.6	3.1 ± 0.6	3.1 ± 0.7	3.1 ± 0.6	0.567	0.762
	Control	3.2 ± 0.4	3.2 ± 0.4	3.1 ± 0.4	3.1 ± 0.3	3.2 ± 0.4		
Temp (°C)	Test	38.4 ± 0.5	38.6 ± 0.5	38.5 ± 0.5	38.6 ± 0.5	38.7 ± 0.5	<0.001^*^	0.107
	Control	38.7 ± 0.4	38.6 ± 0.4	38.7 ± 0.4	38.7 ± 0.5	38.9 ± 0.3		
HR (bpm)	Test	124.8 ± 31.6	122.7 ± 19.9	127.6 ± 27.6	121.0 ± 36.2	122.7 ± 24.6	0.852	0.733
	Control	118.7 ± 21.5	118.6 ± 21.2	120.4 ± 18.0	123.8 ± 22.0	123.3 ± 21.3		
RR (/min)	Test	27.5 ± 13.3	26.6 ± 10.1	27.3 ± 10.0	30.4 ± 10.8	31.0 ± 11.6	<0.001^*^	0.474
	Control	29.6 ± 9.4	29.8 ± 8.1	30.3 ± 8.4	31.0 ± 8.4	32.2 ± 8.3		
BP (mmHg)	Test	136.3 ± 19.6	137.5 ± 25.4	132.9 ± 12.5	130.7 ± 22.1	133.3 ± 18.8	0.152	0.821
	Control	137.0 ± 17.2	132.5 ± 14.4	131.3 ± 13.5	131.3 ± 17.2	130.5 ± 15.0		

Values are presented as mean ± standard deviation. 0 W: Before administration of rcIL-15; 2 W, 4 W, 8 W, 12 W: 2, 4, 8, and 12 weeks after the first administration of rcIL-15, respectively. BCS, Body Condition Score evaluated on a 5-point scale; Temp, temperature; HR, heart rate; RR, respiratory rate; BP, blood pressure. *Statistical significance: *p* < 0.05.

Body weight and BCS remained relatively stable in both groups throughout the 12-week study period, with no significant changes over time (*p* = 0.355 and *p* = 0.567, respectively). However, a significant difference in body weight was noted between the test and control groups (*p* = 0.002), attributed to baseline disparity. There was no significant interaction between time and group, indicating consistent trends across the study period. Body temperature showed a slight increasing trend over time in both groups (*p* < 0.001), but all measurements remained within the normal physiological range. No significant differences were observed between the test and control groups (*p* = 0.107).

Heart rate remained stable throughout the study period in both groups, with no statistically significant changes observed over time (*p* = 0.852) or between groups (*p* = 0.733). This suggests that rcIL-15 did not induce cardiovascular stress or tachycardia. Respiratory rate showed a slight increasing trend over time in both groups (*p* < 0.001). At week 12, the test group recorded a respiratory rate of 31.0 ± 11.6 breaths per minute, while the control group showed 32.2 ± 8.3 breaths per minute. Although an upward trend was observed, respiratory rates remained within normal limits, and no significant differences were found between the groups (*p* = 0.474). These findings indicate that the observed changes were not clinically significant and were likely related to age or other non-treatment-related factors. Blood pressure showed no statistically significant changes over time (*p* = 0.152) or between groups (*p* = 0.821). All measurements remained within normal physiological ranges, suggesting that rcIL-15 administration did not adversely affect cardiovascular function.

Overall, no clinically significant changes were observed in body weight, BCS, body temperature, heart rate, respiratory rate, or blood pressure. Although body temperature and respiratory rate exhibited increasing trends over time, all values remained within normal physiological limits. These results suggest that rcIL-15 administration did not adversely affect the physiological status of dogs with mammary gland tumors.

### 3.3 Inflammatory and thrombotic markers

To assess the anti-inflammatory and antithrombotic effects of rcIL-15, serum levels of C-reactive protein (CRP) and D-dimer were measured. CRP and D-dimer are established biomarkers for systemic inflammation and thrombosis, respectively (17, 18). These markers were evaluated at five time points in 27 dogs from the test group and 28 dogs from the control group (Table 4).

**Table 4:** Changes in vital parameters in test and control groups with mammary gland tumors.

Marker	Groups	0 W	2 W	4 W	8 W	12 W	*p^a^*
CRP (mg/L)	Test	28.35 ± 53.97	7.90 ± 3.81	8.50 ± 4.61	9.61 ± 7.06	10.26 ± 9.70	0.012*
	Control	11.31 ± 10.33	6.54 ± 3.17	6.42 ± 3.16	6.00 ± 3.53	8.46 ± 7.93	0.147
	*p*	0.097	0.130	0.072	0.057	0.463	
D-dimer (ug/mL)	Test	0.29 ± 0.51	0.20 ± 0.21	0.18 ± 0.27	0.20 ± 0.26	0.24 ± 0.71	0.221
	Control	0.14 ± 0.18	0.20 ± 0.34	0.39 ± 1.33	0.11 ± 0.03	0.42 ± 1.13	0.310
	*p*	0.506	0.650	0.589	0.435	0.127	

Values are presented as mean ± standard deviation. 0 W: Before administration of rcIL-15; 2 W, 4 W, 8 W, 12 W: 2, 4, 8, and 12 weeks after the first administration of rcIL-15, respectively. CRP, c-reactive protein. *Statistical significance: *p* < 0.05. ^a^The *p*-values in the rightmost column represent the statistical comparison between values at 0 weeks and 12 weeks within each group.

At baseline, CRP levels were higher in the test group (mean 28.35 mg/L, 95% CI: 7.00–49.70) than in the control group (mean 11.31 mg/L, 95% CI: 7.30–15.32), although this difference was not statistically significant (*p* = 0.097). In the test group, CRP levels decreased steadily over time, reaching within the normal range by week 12. A significant reduction was observed from baseline to week 12 (*p* = 0.012). In contrast, the control group maintained relatively stable CRP concentrations throughout the study period, with no significant changes over time (*p* = 0.147).

D-dimer levels at baseline were 0.29 ± 0.51 μg/mL in the test group and 0.14 ± 0.18 μg/mL in the control group, with no significant difference between groups (*p* = 0.506). D-dimer levels remained within the normal reference range in both groups at all-time points. Although numerically higher values were noted in the control group at weeks 4 and 12, these differences were not statistically significant (all *p* > 0.05).

These results suggest that rcIL-15 may contribute to the reduction of systemic inflammation, as evidenced by decreased CRP levels, while not significantly altering thrombosis-related activity based on D-dimer levels.

### 3.4 Tumor-related biomarkers

**Figure 1:**
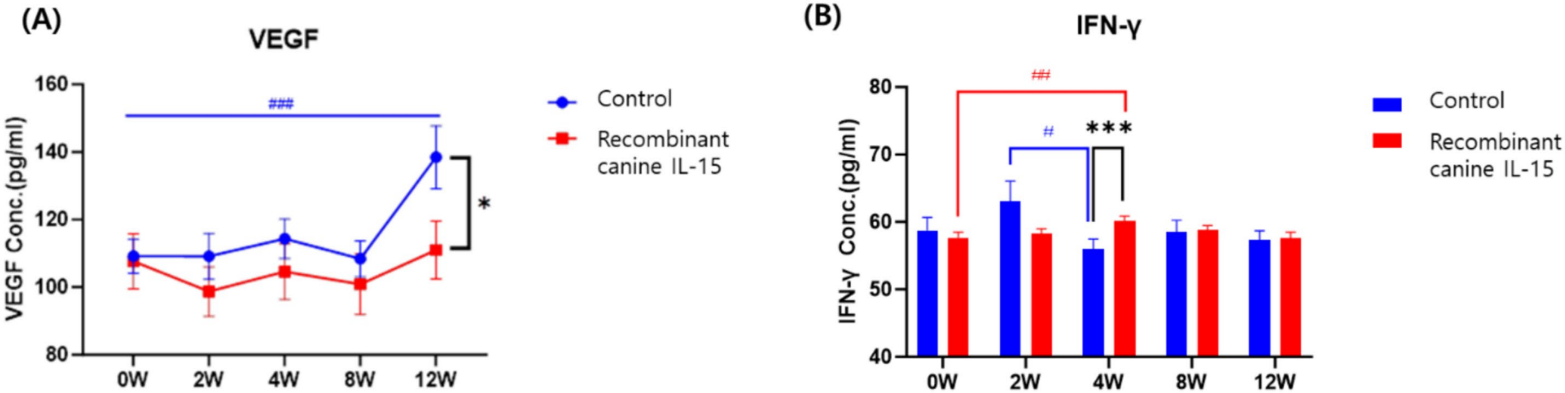
Serum levels of VEGF **(A)** and IFN-*γ* **(B)** in dogs with mammary gland tumors following administration of rcIL-15. VEGF and IFN-γ concentrations were measured in the test group (red box) and control group (blue circle) at baseline (0 W), and at 2, 4, 8, and 12 weeks after treatment. Data are presented as mean ± standard error of the mean (SEM). VEGF, vascular endothelial growth factor; IFN-γ, interferon gamma. Statistical significance between groups at each time point is indicated by asterisks (*p < 0.05, **p < 0.01, ***p < 0.001), while significance within each group compared to baseline (0 W) is denoted by hash symbols (#*p* < 0.05, ##*p* < 0.01, ###*p* < 0.001).

To evaluate the immunomodulatory and antitumor effects of rcIL-15, serum concentrations of vascular endothelial growth factor (VEGF) and interferon-gamma (IFN-*γ*) were measured at baseline (0 W) and at 2, 4, 8, and 12 weeks after treatment (Figure 1). A total of 51 dogs (test group: *n* = 25, control group: *n* = 26) provided sufficient serum samples for analysis. Values were obtained using a sandwich ELISA and analyzed both as absolute concentrations and relative changes from baseline.

At baseline, VEGF levels were not significantly different between groups (*p* = 0.082). Over time, the test group showed relatively stable VEGF levels, with no significant increase from baseline to week 12 (0 W: 107.7 ± 42.3 pg./mL; 12 W: 111.0 ± 44.4 pg./mL; *p* = 0.729). In contrast, the control group exhibited a significant increase in VEGF levels during the same period (0 W: 109.2 ± 26.7 pg./mL; 12 W: 138.5 ± 49.1 pg./mL; *p* = 0.000). At week 12, VEGF levels were significantly lower in the test group compared to the control group (*p* = 0.037). This suggests that rcIL-15 may suppress VEGF upregulation associated with tumor progression, thereby limiting angiogenesis and potentially impeding tumor growth.

For IFN-*γ*, baseline concentrations were similar between the two groups (*p* = 0.219). In the test group, IFN-γ levels significantly increased from baseline to week 4 (57.7 ± 4.2 pg./mL to 60.1 ± 3.8 pg./mL; *p* = 0.002), while the control group showed a significant decrease during the same interval (58.7 ± 10.4 pg./mL to 55.9 ± 7.8 pg./mL; *p* = 0.012). Group comparison at week 4 also revealed significantly higher IFN-*γ* levels in the test group than in the control group (*p* = 0.001). These findings indicate that rcIL-15 effectively enhanced IFN-γ secretion, which is associated with antitumor immune activation.

Together, these results suggest that rcIL-15 exerts dual effects on tumor progression: it reduces pro-angiogenic signaling by inhibiting VEGF elevation and simultaneously boosts antitumor immune responses through IFN-γ stimulation. These effects likely contribute to the observed therapeutic benefits in dogs with mammary gland tumors.

### 3.5 Quality of life and clinical sign assessment

Owner-reported QoL was evaluated using a validated questionnaire consisting of 29 items across 10 categories, including happiness, mental health, pain, appetite, hygiene, hydration, mobility, fatigue, cardiopulmonary status, and overall well-being. Assessments were conducted at baseline and at 2, 4, 8, and 12 weeks post-treatment (Supplementary Table 1). At baseline, the test group showed lower QoL scores in several domains compared to the control group, particularly in sleep duration, tumor-associated pain, and appetite. However, over the 12-week period, the test group demonstrated significant improvements in multiple categories. Significant between-group differences favoring the test group were observed in happiness (*p* = 0.029), mental health (*p* = 0.031), and pain relief (*p* = 0.017). Improvements in the control group were limited to mental health and hygiene. Importantly, significant time-by-group interactions were detected in tumor pain (*p* < 0.001) and difficulty rising (*p* = 0.013), indicating a more pronounced improvement in the test group over time. *Post hoc* comparisons confirmed that pain scores significantly decreased at all-time points in the test group compared to baseline, whereas no such trend was observed in the control group. By week 12, most QoL parameters improved in the test group to levels comparable with or exceeding those of the control group. These findings suggest that rcIL-15 not only helped alleviate clinical symptoms but also positively influenced overall quality of life in dogs with mammary gland tumors from the owner’s perspective.

Veterinarians assessed clinical signs, including behavioral changes, gastrointestinal signs, respiratory signs, and neurological signs. The test group showed greater improvement in behavioral signs and gastrointestinal disturbances compared to the control group. Respiratory signs, such as coughing and dyspnea, increased significantly in the control group by week 12, whereas the test group maintained stable respiratory function. No neurological signs were observed in either group.

These findings suggest that rcIL-15 not only improves the quality of life but also positively impacts clinical signs, supporting its therapeutic potential in dogs with mammary gland tumors.

### 3.6 Hematological and biochemical safety evaluations

To evaluate the safety of rcIL-15, hematological, biochemical, and electrolyte parameters were monitored at baseline and at 2, 4, 8, and 12 weeks post-treatment. Complete blood counts (CBCs) revealed a mild decrease in total white blood cell (WBC) counts in both groups, primarily due to a slight reduction in neutrophils, while lymphocyte counts showed a modest increase. Other differential counts, including monocytes, eosinophils, and basophils, remained stable, and no significant differences were observed between groups. Red blood cell (RBC) counts, hemoglobin (Hb), hematocrit (HCT), and platelet (PLT) counts remained within normal ranges throughout the study. A transient elevation in HCT and Hb was observed in the control group at week 8, possibly associated with gastrointestinal disturbances, but resolved by week 12. No hematologic abnormalities were attributed to rcIL-15 administration. Serum chemistry analysis revealed no significant abnormalities in glucose, renal markers (BUN, creatinine), calcium, phosphorus, or total protein levels. While the test group exhibited higher baseline levels of ALKP and GGT, indicative of possible pre-existing liver conditions, these values showed a declining trend over time with no post-treatment elevations, suggesting no hepatotoxicity associated with rcIL-15. Electrolyte concentrations, including sodium, potassium, and chloride, remained stable and within normal physiological ranges in both groups, indicating that rcIL-15 administration did not disrupt electrolyte balance or metabolic function. Overall, the results confirm that rcIL-15 is well tolerated without adverse hematological, biochemical, or electrolyte effects in dogs with mammary gland tumors.

### 3.7 Adverse events and safety profile

Adverse events were monitored throughout the study period to evaluate the safety and tolerability of rcIL-15. The most commonly reported side effect was mild gastrointestinal disturbance, including vomiting and diarrhea, which were transient and resolved without intervention. Injection site reactions, characterized by mild swelling or redness, were reported in two cases and were managed with supportive care. A single case of genital irritation was noted and resolved without further complications.

No severe adverse events necessitating discontinuation of rcIL-15 were observed. All hematological and blood chemistry parameters remained within normal ranges, with no significant changes in liver or kidney function markers. Electrolyte levels also showed no significant variations. These findings confirm the safety profile of rcIL-15 when administered to dogs with mammary gland tumors.

All observed adverse events were mild and manageable with supportive care, indicating a favorable safety profile. No treatment-related mortality or severe systemic toxicity was observed. The safety assessments, including hematological, biochemical, and electrolyte analyses, showed no significant abnormalities in either group. The absence of significant adverse effects supports the clinical safety of rcIL-15 in dogs with mammary gland tumors.

## 4 Discussion

This study investigated the safety and therapeutic potential of rcIL-15 as an adjunct to surgical excision in dogs with MGTs. The results demonstrate that rcIL-15 is well tolerated and may offer clinical benefits, including improvement in clinical signs and QoL. These findings add to the growing evidence supporting the use of immunotherapy in veterinary oncology.

The safety profile of rcIL-15 was carefully evaluated in this study, particularly in the context of dogs with MGTs. Over the 12-week observation period, no significant changes were noted in physiological parameters or in hematological, biochemical, or electrolyte values, indicating that rcIL-15 was well tolerated. These findings are in line with earlier preclinical and clinical studies demonstrating the favorable safety of rcIL-15 in both healthy and cancer-bearing dogs (12, 19). Importantly, no signs of severe systemic toxicity or cytokine release syndrome (CRS)—which has been documented in dogs treated with adoptive cell therapy and IL-2-based protocols (20, 21)—were observed in this study. This favorable safety profile distinguishes rcIL-15 from other proinflammatory cytokine-based immunotherapies. The most common adverse events included mild gastrointestinal disturbances and transient injection site reactions, all of which resolved without intervention. Similar low-grade toxicities have been reported in prior studies where rcIL-15 was used either alone or in combination with metronomic cyclophosphamide (19), further supporting its suitability for use in geriatric canine patients and its potential as a long-term immunomodulatory therapy.

In addition to its safety profile, rcIL-15 was associated with notable improvements in clinical symptoms and QoL. Owner-reported assessments indicated meaningful improvements particularly in pain control, appetite, activity level, and overall well-being, with the most prominent changes observed between weeks 4 and 8 of treatment. Although QoL evaluation in veterinary medicine inherently relies on subjective observations, this study employed validated questionnaires to minimize bias (14, 22). These improvements are likely attributable to alleviation of tumor-associated inflammation and discomfort, both of which are common in dogs with advanced MGTs (4). A previous pilot study similarly reported improved well-being in dogs treated with rcIL-15, suggesting that its therapeutic benefits may extend beyond tumor control to alleviation of tumor-related clinical signs (19). Nevertheless, the absence of objective activity monitoring or clinician-based scoring remains a limitation, underscoring the need for more structured outcome measures in future studies.

In parallel with symptomatic improvement, the observed reduction in serum CRP levels in the rcIL-15 group may reflect a decrease in systemic inflammation. Elevated CRP has been linked with advanced disease stage and poor prognosis in both human and canine cancers, including lymphoma and solid tumors (23, 24). Although the anti-inflammatory role of IL-15 is not fully elucidated in veterinary species, previous studies have suggested that IL-15 can indirectly reduce inflammatory cytokines through immune modulation, supporting its potential to alleviate tumor-associated inflammation (25, 26).

In addition to its anti-inflammatory potential, rcIL-15 administration was associated with favorable immunological changes that may contribute to its antitumor effects. Notably, VEGF levels remained stable in the rcIL-15 group while significantly increasing in controls, suggesting that rcIL-15 may exert indirect anti-angiogenic activity. Elevated VEGF expression has been linked to increased malignancy and metastasis in canine MGTs (27), and its suppression is considered a valuable therapeutic goal in oncology. Although this study did not include histological vascular assessments, the VEGF stabilization aligns with previous reports in murine models where IL-15 administration led to reduced tumor vascularization (28). Alongside this, a marked increase in IFN-*γ* levels was observed in the test group, consistent with the well-documented ability of IL-15 to activate NK and CD8+ T cells. These cell types play a pivotal role in immune-mediated tumor surveillance, and their activation is reflected in elevated IFN-γ secretion. Prior canine studies have confirmed that rcIL-15 enhances NK and CD8 + T cell populations and increases Ki-67 expression, indicative of lymphocyte proliferation (12). While IFN-γ elevation alone may not confirm direct cytotoxicity, its presence supports the immunostimulatory role of IL-15 and may indicate early immune activation. Similar immune activation trends were reported in a phase I clinical trial using inhaled recombinant human IL-15 in dogs with metastatic melanoma or osteosarcoma, where cytokine induction and increased PBMC cytotoxicity correlated with clinical benefit. These findings support the broader immunostimulatory potential of IL-15–based therapies across different tumor types and delivery methods (29). However, interpretation of these immune trends should be made with caution, as the study did not include cellular-level analyses such as flow cytometry or tumor-infiltrating lymphocyte profiling. The absence of such data limits the ability to draw mechanistic conclusions regarding immune cell dynamics.

Interpretation of the findings should take several limitations into account. The 12-week follow-up period, while sufficient for capturing short-term safety and clinical responses, was not long enough to evaluate recurrence, metastasis, or overall survival. Baseline variability in serum CRP concentrations—particularly the higher average value in the test group—may have influenced the interpretation of anti-inflammatory trends. The study population included dogs with histologically diverse mammary gland tumors, but consistent grading data were not available, which limited subgroup analysis based on tumor grade. Additionally, although group assignment was randomized, a small imbalance in breed and body size distribution between groups may have affected comparisons of physiological parameters. Differences in age between groups, although not controlled for statistically, may also have influenced treatment outcomes and owner-reported quality of life assessments.

Despite these limitations, the findings support the continued investigation of rcIL-15 in canine solid tumors. The results highlight the potential of rcIL-15 to improve quality of life and modulate systemic inflammation and tumor-related immune responses when used as an adjunct to surgery. Future studies incorporating longer follow-up, objective functional assessments, and in-depth immune profiling are necessary to validate and expand upon these observations. In particular, prospective survival studies will be essential to assess long-term therapeutic outcomes, and mechanistic investigations involving immune cell phenotyping and analysis of the tumor microenvironment may help clarify the pathways through which rcIL-15 exerts its effects. In addition, stratified randomization or statistical adjustment for baseline variables such as age may help improve the interpretability of treatment effects in future trials.

## 5 Conclusion

In dogs with mammary gland tumors, rcIL-15 administered as an adjunct to surgical treatment was safe, well tolerated, and associated with improvements in quality of life and systemic inflammation. The observed reduction in CRP, stabilization of VEGF, and increase in IFN-*γ* levels suggest that rcIL-15 may exert complementary anti-inflammatory and immunostimulatory effects relevant to tumor control. While these findings support its use as a supportive immunotherapeutic strategy in veterinary oncology, their clinical significance requires validation in long-term, prospective studies. Further research should also aim to define optimal dosing strategies and evaluate rcIL-15 in combination with other therapeutic modalities, alongside mechanistic investigations to better understand its immunological effects.

## Data Availability

The original contributions presented in the study are included in the article/Supplementary material, further inquiries can be directed to the corresponding author.
