# Supplementary material for: Safety and efficacy of canine recombinant IL-15 in mammary gland tumors
Source: Front Vet Sci. 2025 Jun 9;12:1603421. doi: 10.3389/fvets.2025.1603421 (PMC12183958; doi:10.3389/fvets.2025.1603421)
Supplement: Supplementary file 1 [file Table_1.DOCX]

**Supplemented Table 1. Owner-assessed quality of life in test and control groups with mammary gland tumors**

| Questionnaires | Groups | 0W | 2W | 4W | 8W | 12W | *p*  (time) | *p*  (group) |
| --- | --- | --- | --- | --- | --- | --- | --- | --- |
| **Joy of life** (0: Never, 1: Infrequently, 2: Sometimes, 3: Frequently, 4: Always) | | | | | | | | |
| My pet has been playing a normal amount for him/her | **Test** | 2.8±1.1 | 3.2±0.9 | 3.3±0.8 | 3.1±0.9 | 3.3±0.9 | 0.008* | 0.875 |
|  | **Control** | 2.9±1.4 | 3.2±0.8 | 3.2±1.2 | 3.3±1.0 | 3.0±1.0 |  |  |
| My pet has been responding to my presence | **Test** | 3.4±0.9 | 3.4±0.6 | 3.5±0.8 | 3.5±1.0 | 3.6±0.8 | 0.302 | 0.681 |
|  | **Control** | 3.4±0.8 | 3.2±1.0 | 3.3±1.2 | 3.5±0.8 | 3.4±1.1 |  |  |
| My pet has been enjoying life | **Test** | 2.7±1.2 | 3.0±1.1 | 3.0±1.0 | 3.0±1.0 | 3.0±1.1 | 0.064 | 0.872 |
|  | **Control** | 2.7±1.4 | 2.8±1.2 | 3.0±1.3 | 3.1±1.3 | 3.1±1.3 |  |  |
| My pet has been happy to see me when I get home | **Test** | 3.5±1.0 | 3.5±1.0 | 3.4±0.9 | 3.5±1.0 | 3.6±0.8 | 0.992 | 0.540 |
|  | **Control** | 3.2±1.1 | 3.2±1.0 | 3.4±1.2 | 3.3±1.1 | 3.5±0.7 |  |  |
| **Mental status** (0: Strongly disagree, 1: Disagree, 2: Neutral, 3: Agree, 4: Strongly agree) | | | | | | | | |
| My pet has had more good days than bad days | **Test** | 3.2±0.8 | 3.2±0.8 | 3.1±0.7 | 3.0±0.9 | 3.1±1.1 | 0.176 | 0.685 |
|  | **Control** | 2.7±1.3 | 3.1±0.8 | 3.5±0.6 | 3.3±0.8 | 3.3±0.9 |  |  |
| My pet has been sleeping more than usual | **Test** | 2.5±1.2 | 2.2±1.2 | 1.5±1.0 | 2.0±1.2 | 1.9±0.9 | <0.001* | 0.005* |
|  | **Control** | 1.6±1.3 | 1.4±1.1 | 1.1±1.0 | 1.4±1.4 | 1.3±1.2 |  |  |
| My pet has seemed depressed | **Test** | 1.2±1.0 | 1.1±1.0 | 1.0±0.9 | 1.0±0.9 | 1.0±0.9 | 0.004* | 0.264 |
|  | **Control** | 1.1±1.2 | 1.0±0.9 | 0.6±0.7 | 0.9±1.1 | 0.6±0.8 |  |  |
| My pet has seemed anxious/stressed | **Test** | 1.2±0.9 | 1.2±0.9 | 1.0±1.0 | 1.0±0.9 | 1.0±0.9 | 0.122 | 0.066 |
|  | **Control** | 1.0±0.9 | 0.8±0.7 | 0.5±0.7 | 1.0±1.2 | 0.7±0.8 |  |  |

*(Continue)*

| Questionnaires | Groups | 0W | 2W | 4W | 8W | 12W | *p*  (time) | *p*  (group) |
| --- | --- | --- | --- | --- | --- | --- | --- | --- |
| **Pain** (0: Never, 1: Infrequently, 2: Sometimes, 3: Frequently, 4: Always) | | | | | | | | |
| My pet has been in pain | **Test** | 0.6±0.8 | 0.5±1.0 | 0.1±0.3 | 0.4±1.0 | 0.3±0.6 | 0.126 | 0.119 |
|  | **Control** | 0.1±0.5 | 0.3±0.9 | 0.1±0.4 | 0.3±0.9 | 0.1±0.4 |  |  |
| My pet has appeared restless | **Test** | 0.2±0.5 | 0.4±1.0 | 0.1±0.3 | 0.1±0.5 | 0.2±0.5 | 0.689 | 0.573 |
|  | **Control** | 0.1±0.5 | 0.1±0.4 | 0.2±0.5 | 0.2±0.6 | 0.2±0.6 |  |  |
| My pet has seemed painful in the tumor area | **Test** | 0.8±1.2 | 0.3±0.9 | 0.1±0.3 | 0.2±0.5 | 0.2±0.5 | <0.001* | 0.002* |
|  | **Control** | 0.1±0.3 | 0.0±0.0 | 0.0±0.0 | 0.0±0.0 | 0.0±0.0 |  |  |
| **Appetite/food intake** (0: Never, 1: Infrequently, 2: Sometimes, 3: Frequently, 4: Always) | | | | | | | | |
| My pet has been eating a normal amount | **Test** | 3.0±1.1 | 3.3±0.7 | 3.3±0.8 | 3.0±1.4 | 3.6±0.7 | 0.161 | 0.037* |
|  | **Control** | 3.7±0.6 | 3.6±0.6 | 3.7±0.6 | 3.5±0.9 | 3.6±0.6 |  |  |
| My pet has shown a capricious appetite | **Test** | 0.8±1.0 | 0.8±1.2 | 0.5±0.9 | 0.8±1.2 | 0.6±0.7 | 0.307 | 0.056 |
|  | **Control** | 0.5±0.8 | 0.3±0.6 | 0.2±0.4 | 0.5±0.9 | 0.4±0.7 |  |  |
| My pet has been eating his/her usual diet | **Test** | 3.0±1.2 | 3.3±0.7 | 3.3±0.9 | 3.2±1.2 | 3.5±0.9 | 0.632 | 0.043* |
|  | **Control** | 3.7±0.7 | 3.5±0.9 | 3.6±0.9 | 3.8±0.6 | 3.9±0.3 |  |  |
| My pet has shown difficulty in eating | **Test** | 0.3±0.7 | 0.6±1.1 | 0.3±0.6 | 0.2±0.5 | 0.4±0.9 | 0.668 | 0.200 |
|  | **Control** | 0.0±0.2 | 0.1±0.3 | 0.0±0.0 | 0.2±0.8 | 0.2±0.8 |  |  |
| Hygiene (0: Never, 1: Infrequently, 2: Sometimes, 3: Frequently, 4: Always) | | | | | | | | |
| My pet has been keeping himself/herself clean | **Test** | 2.6±1.0 | 2.7±1.1 | 3.0±0.9 | 2.9±1.3 | 3.0±1.0 | 0.002* | 0.202 |
|  | **Control** | 2.2±1.1 | 2.5±1.1 | 2.5±1.3 | 2.8±1.1 | 3.0±1.1 |  |  |
| My pet’s coat has been in good condition | **Test** | 2.7±1.1 | 2.8±1.2 | 2.9±1.1 | 3.0±1.2 | 3.0±1.0 | 0.009* | 0.703 |
|  | **Control** | 2.4±1.2 | 2.7±1.1 | 3.2±1.1 | 2.8±1.0 | 3.0±1.1 |  |  |

*(Continue)*

| Questionnaires | Groups | 0W | 2W | 4W | 8W | 12W | *p*  (time) | *p*  (group) |
| --- | --- | --- | --- | --- | --- | --- | --- | --- |
| **Hydration status** (0: Never, 1: Infrequently, 2: Sometimes, 3: Frequently, 4: Always) | | | | | | | | |
| My pet has been drinking a normal amount | **Test** | 3.4±0.7 | 3.4±0.8 | 3.5±0.7 | 3.5±0.9 | 3.4±0.8 | 0.680 | 0.061 |
|  | **Control** | 3.4±0.8 | 3.6±0.6 | 3.7±0.6 | 3.7±0.6 | 3.7±0.5 |  |  |
| My pet has been urinating normally | **Test** | 3.2±1.0 | 3.3±0.9 | 3.3±1.0 | 3.1±1.2 | 3.3±1.1 | 0.876 | 0.013* |
|  | **Control** | 3.8±0.5 | 3.7±0.6 | 3.7±0.6 | 3.7±0.6 | 3.8±0.6 |  |  |
| **Mobility** (0: Never, 1: Infrequently, 2: Sometimes, 3: Frequently, 4: Always) | | | | | | | | |
| My pet has been moving around normally | **Test** | 3.4±0.7 | 3.3±0.9 | 3.5±0.9 | 3.5±0.7 | 3.4±0.7 | 0.968 | 0.280 |
|  | **Control** | 3.5±0.7 | 3.7±0.6 | 3.5±0.8 | 3.5±0.8 | 3.5±0.7 |  |  |
| My pet has shown difficulties in getting up | **Test** | 0.3±0.7 | 0.4±0.6 | 0.3±0.6 | 0.8±1.4 | 0.7±1.3 | 0.793 | 0.886 |
|  | **Control** | 0.6±1.2 | 0.9±1.5 | 0.8±1.4 | 0.4±1.1 | 0.4±1.1 |  |  |
| My pet’s activity level has been normal for  him/her | **Test** | 3.0±1.0 | 3.4±0.9 | 3.4±0.9 | 3.1±1.0 | 3.3±0.8 | 0.588 | 0.304 |
|  | **Control** | 3.3±1.2 | 3.5±0.9 | 3.2±1.3 | 3.5±0.8 | 3.4±0.9 |  |  |

*(Continue)*

| Questionnaires | Groups | | 0W | 2W | 4W | 8W | 12W | *p*  (time) | *p*  (group) |
| --- | --- | --- | --- | --- | --- | --- | --- | --- | --- |
| **Cardiovascular/respiratory system** (0: Never, 1: Infrequently, 2: Sometimes, 3: Frequently, 4: Always) | | | | | | | | | |
| My pet’s breathing has been normal | | **Test** | 3.7±0.6 | 3.5±1.0 | 3.7±0.6 | 3.5±0.9 | 3.6±0.8 | 0.425 | 0.115 |
|  |  | **Control** | 3.9±0.5 | 3.8±0.5 | 3.8±0.5 | 3.8±0.5 | 3.8±0.5 |  |  |
| My pet has been getting tired easily | | **Test** | 1.3±1.2 | 1.3±1.3 | 0.9±1.0 | 1.0±1.3 | 0.9±1.0 | 0.211 | 0.035* |
|  |  | **Control** | 0.7±1.3 | 0.7±1.0 | 0.7±1.1 | 0.7±1.0 | 0.5±1.0 |  |  |
| My pet has shown coughing | | **Test** | 0.7±1.1 | 0.4±0.9 | 0.4±0.8 | 0.7±1.0 | 0.4±0.8 | 0.607 | 0.787 |
|  |  | **Control** | 0.5±1.1 | 0.7±1.2 | 0.3±0.7 | 0.5±0.9 | 0.6±1.1 |  |  |
| General health (0: Strongly disagree, 1: Disagree, 2: Neutral, 3: Agree, 4: Strongly agree) | | | | | | | | | |
| My pet has generally been well | | **Test** | 3.3±0.7 | 3.3±0.7 | 3.5±0.7 | 3.5±0.7 | 3.3±0.9 | 0.086 | 0.946 |
|  |  | **Control** | 3.4±0.7 | 3.3±0.8 | 3.6±0.5 | 3.5±0.6 | 3.5±0.5 |  |  |
| Did the QoL of my pet drastically worsen after the  tumor diagnosis | | **Test** | 1.1±0.9 | 1.1±1.1 | 0.5±0.8 | 0.9±1.2 | 1.0±1.0 | 0.001* | 0.040* |
|  |  | **Control** | 0.6±0.7 | 0.6±0.9 | 0.4±0.7 | 0.5±0.7 | 0.6±0.6 |  |  |
| Did the QoL of my pet drastically worsen after  chemotherapy? | | **Test** | 1.0±0.7 | 0.8±0.8 | 0.7±0.9 | 0.7±0.8 | 0.6±0.8 | 0.426 | 0.036* |
|  |  | **Control** | 0.4±0.6 | 0.4±0.6 | 0.3±0.6 | 0.4±0.8 | 0.5±0.8 |  |  |
| My pet has been having a good QoL | | **Test** | 3.0±0.9 | 3.0±0.9 | 3.2±0.9 | 3.1±0.9 | 3.0±0.9 | 0.371 | 0.457 |
|  |  | **Control** | 2.7±1.4 | 2.7±1.2 | 3.1±1.2 | 3.0±1.1 | 2.9±1.2 |  |  |

Values are presented as mean ± standard deviation.
0W: Before administration of recombinant canine IL-15; 2W, 4W, 8W, 12W: 2, 4, 8, and 12 weeks after the first administration of recombinant canine IL-15, respectively.
*Statistical significance: *p* < 0.05.
